# Noise exposure during robot-assisted total knee arthroplasty

**DOI:** 10.1007/s00402-022-04454-w

**Published:** 2022-05-04

**Authors:** Tim Hönecke, Michael Schwarze, Matthias Wangenheim, Peter Savov, Henning Windhagen, Max Ettinger

**Affiliations:** 1 Department of Orthopedic Surgery, Hannover Medical School, Anna-von-Borries-Str. 1-7, 30625 Hannover, Germany; 2 Laboratory for Biomechanics and Biomaterials, Hannover Medical School, 30625 Hannover, Germany; 3 Institute of Dynamics and Vibrations, Leibniz University Hannover, Welfengarten 1, 30167 Hannover, Germany; 4 Hahnenstraße 13, 30167 Hannover, Germany

**Keywords:** Total knee arthroplasty, Noise-inducing hearing loss, Gonarthrosis, Computer-assisted surgery, Robot-assisted surgery occupational noise

## Abstract

The aim of the study was to examine the noise exposure for operating theater staff during total knee arthroplasty (TKA) with three different robot systems. There is already evidence that noise exposure during TKA performed manually exceeds recommended guidelines for occupational noise. Therefore, if surgical staff is exposed to it for several years, the development of noise-inducing hearing loss (NIHL) is significantly increased. To investigate the noise exposure during robot-assisted TKA, the study measured the average noise and the peak sound pressure during TKA with MAKO robot (Stryker, Kalamazoo, Michigan, United States), NAVIO robot (Smith and Nephew, London, Great Britain), and CORI robot (Smith and Nephew, London, Great Britain) using a class 1 sound level meter. Each robot system exceeds the recommended guidelines from the national institute for occupational safety and health. While the MAKO robot had the highest average sound level (93.18 dB(A)) of the three robot systems (NAVIO: 88.88 dB(A), CORI: 89.38 dB(A)), the peak sound level was the highest with the NAVIO Robot (134.48 dB(C)) compared to the MAKO Robot (128.98 dB(C)) and CORI robot (126.48 dB(C)). Robot-assisted TKA is a risk factor for NIHL, like manually performed TKA. Further research for decreasing the noise exposure during TKA is needed to minimize the hearing loss in operating theater staff.

## Introduction

Nowadays, symptomatic gonarthrosis is a common disease among the global population [1]. The replacement of a joint by a total knee arthroplasty (TKA) presents a good therapeutic option, which led to an immense increase in knee endoprosthesis in recent years. Given the enhancing surgery methods [2], a continuation of that steady growth is expected for the future.

To improve the postoperative functional outcome of the TKA and patient satisfaction, a recent innovation is the use of computer-assisted and robot-assisted navigation systems for the implantation of the endoprosthesis. This development resulted in a steady growth of computer-assisted and robot-assisted TKA within the last years [3–5]. The TKA procedure requires the use of high-powered instruments, such as the surgical saw or mallet, which generate significant noise. Being exposed to this increased operative noise can be harmful [6, 7]. Therefore, surgical staff, who have been directly exposed to this noise for several years, are particularly in danger of incurring substantial health consequences such as noise-inducing hearing loss (NIHL), as Willet et al. have shown [8]. They found a significant hearing loss among 50% of the staff who worked in the operating theater for several years [8].

In addition, new studies stress that the noise levels in operating theaters generated during manual total joint replacements are above the recommended guidelines for occupational noise set by the National Institute for occupational safety and health (NIOSH) [9, 10]. According to NIOSH, the average occupational noise (LAeq) should not be louder than 85 decibels in an A-weighted scale (dB(A)) over a period of 8 h, as well as a ceiling limit of 140 dB for peak sound pressure (LCpeak) [11]. National guidelines from England and Germany also clearly recommend that the daily noise average should not exceed 85 dB. Moreover, the maximum sound level during work should not exceed 137 dB [12, 13].

The studies mentioned above only measure the noise level of manually performed knee and hip joint endoprostheses without using a robotic surgical device. For computer-navigated surgeries, the robotic device could be an additional noise source. So far, there is a lack of data examining the sound level of a computer-navigated and robotic-assisted total knee arthroplasty. This study aims to measure the surgeon’s average and maximum noise exposure during robotic-assisted implantation of a knee endoprosthesis to derive potential health consequences. Our primary hypothesis is that the use of robotic surgical devices exceeds NIOSH recommendations for occupational noise.

## Materials and methods

This study measured noise exposure during the performance of TKA with three different robotic surgical devices:

- TKA with MAKO robot (Stryker, Kalamazoo, Michigan, United States)
- TKA with NAVIO robot (Smith and Nephew, London, Great Britain)
- TKA with CORI robot (Smith and Nephew, London, Great Britain)

The CORI robot is the next generation of robot systems from Smith and Nephew and the evolution of the NAVIO robot.

### Surgery and saw blades

All surgeries were performed using a medial parapatellar approach. During TKA with MAKO Robot, the femoral and tibial bone cuts were performed using a fully oscillating standard Mako saw blade (thickness: 2 mm, width 16 mm) or a narrow saw blade (thickness 2 mm and width 9 mm).

In surgeries with NAVIO Robot, the distal femoral cut was conducted using the reamer of the NAVIO handpiece. Subsequently, the remaining femoral and tibial bone cuts were performed manually using the Stryker S8 handpiece and a Smith and Nephew saw blade for Stryker machines (thickness: 1.37 mm, width 19 mm). The standard saw block for the Journey knee prosthesis from Smith and Nephew was used.

During surgeries with CORI robot, the distal femoral cut was conducted with the reamer of the CORI handpiece. The other surgical steps were similar to the procedure with the NAVIO robot using the same instruments.

### Noise level measurement

The sound level meter XL2 (NTi Audio, Schaan, Liechtenstein) was used to measure the operating theater's noise level at a range of 50–150 dB. These measurements were only conducted in surgeries with patients suffering primary gonarthrosis, while diseases indicative of decreased bone mass, such as osteoporosis, Paget’s disease, or rheumatoid arthritis, were excluded.

The measurement device was placed 1.5 m from the surgical site using a microphone stand to ensure sterility. During surgery, care was taken to ensure that no objects or people were between the measurement device and the noise source.

The measurement started at the beginning of the incision time and ended when the skin closure was completed. Data were logged every 30 s. An A-weighted scale (dB(A)) and a C-weighted scale (dB(C)) were measured parallelly. The A-weighted scale considers the frequency-dependent sensitivity of the human ear and is used to calculate an equivalent continuous sound level with an averaging timeframe of 30 min (LAeq). The C-weighted scale is used to evaluate peak sound pressure levels at a time interval of 30 s (LCpeak).

### Sound level calculation

To represent the surgeon’s exposure as accurately as possible, the sound level arriving at the surgeon’s ear was calculated using the following formula:

$${L}_{\mathrm{OP}}={L}_{\mathrm{Meas}}+20*\mathrm{log}\left(\frac{{r}_{2}}{{r}_{1}}\right),$$

where *L*_OP_ is the sound level at the surgeon’s ear, *L*_Meas_ is the sound level at the measuring location, *r*_2_ is the distance of the measuring device from the point sound source = 150 cm, and r_1_ is the distance of the surgeon’s ear from the point source = 40 cm.

Formula 1.1: Sound level calculation for the noise exposure of the surgeon’s ear.

### Statistical analysis

For LAeq and LCpeak, the maximum occurring value was determined during each surgery. The differences between groups were evaluated using the non-parametric Mann–Whitney *U* test with a significance level of 5%.

## Results

The present study measured the noise level of 21 robotic-assisted total knee arthroplasties in total. Eight surgeries were performed with the MAKO robot, seven were completed by the NAVIO robot, and six were measured in surgeries with the CORI robot.

**Fig. 1:**
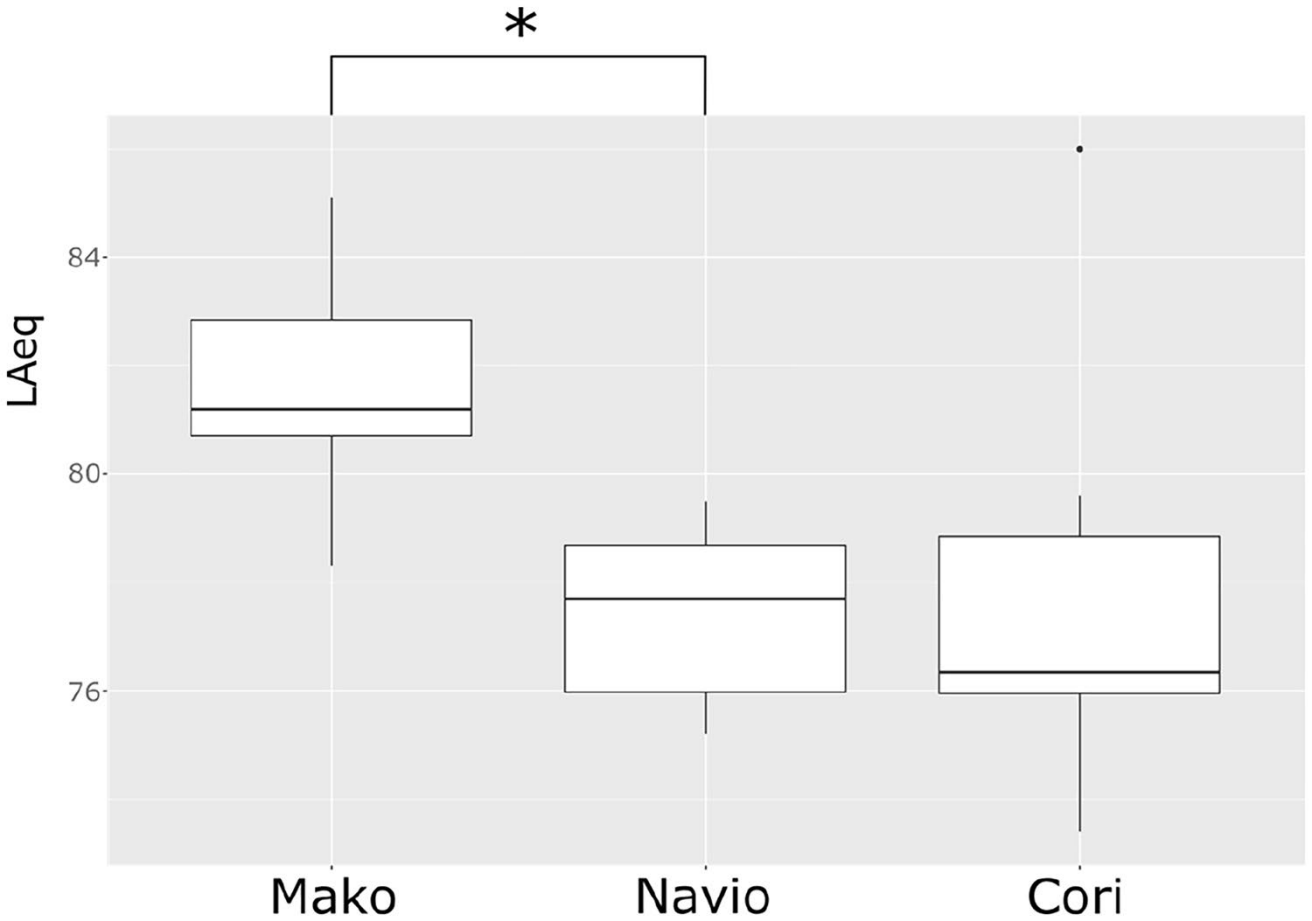
Equivalent continuous sound level (LAeq) of surgery with MAKO, NAVIO and CORI robot. *Significant difference (*p* = 0.05)

**Fig. 2:**
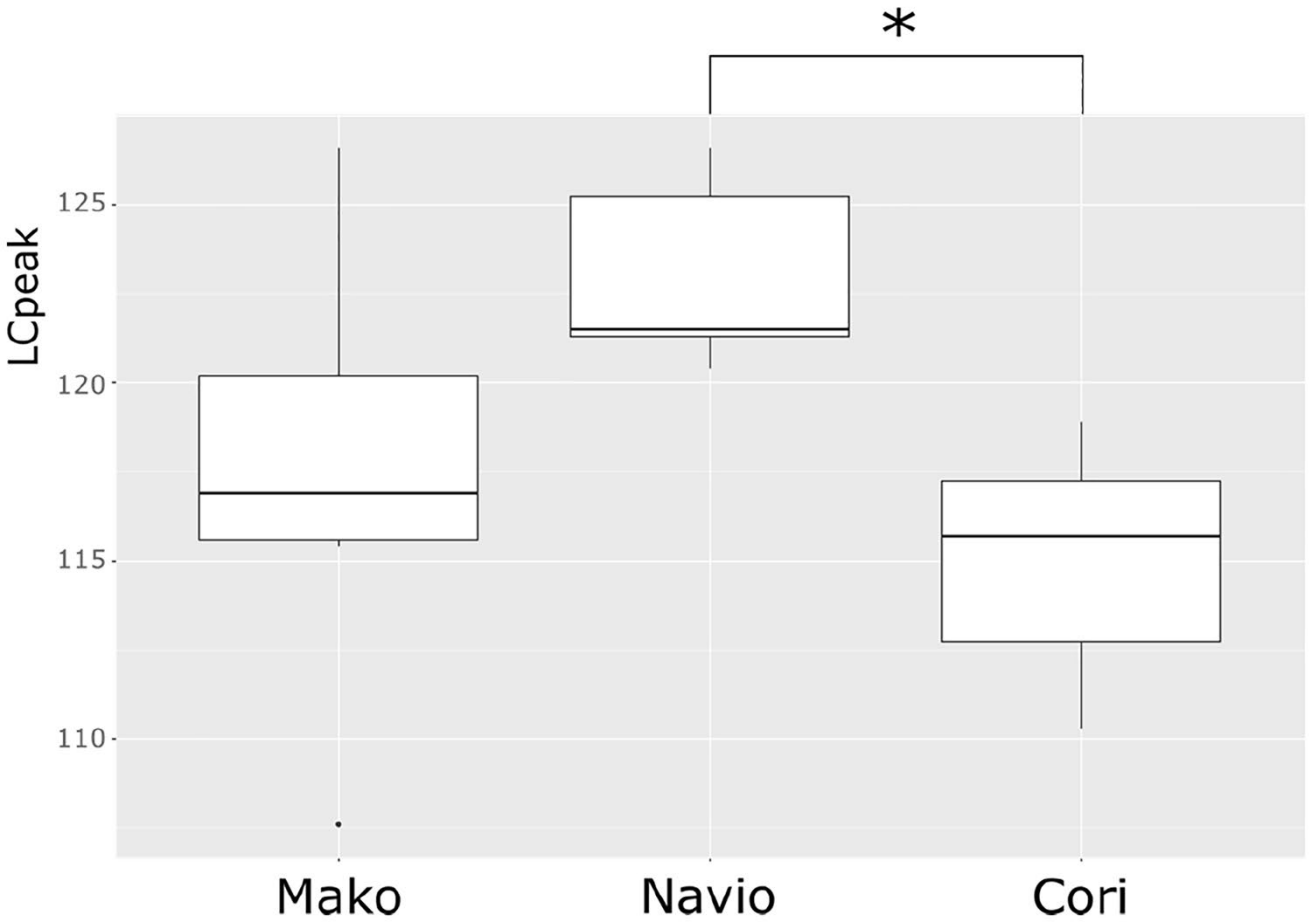
Peak sound pressure levels (LC_peak_) of surgery with MAKO, NAVIO and CORI robot. *Significant difference (*p* = 0.05)

The average sound level LAeq was 81.7 dB(A) when using the MAKO robot compared to 77.4 dB(A) with the NAVIO robot (*p* = 0.008) and 77.9 dB(A) with the CORI robot (*p* = 0.073). Unlike the average sound level, LCpeak was the highest during TKA using a NAVIO robot with 123.0 dB(C) compared to the MAKO robot with 117.5 dB(C) (*p* = 0.0733) and the CORI robot with 115.0 dB(C) (*p* = 0.005) (Figs. 1, 2).

When calculating the LA_eq_ of the surgeon’s ear with the formula 1.1, the resulting sound level is 93.18 dB(A) when using the MAKO robot compared to 88.88 dB(A) when using the NAVIO robot and 89.38 dB(A) using the CORI robot. When calculating LC_peak_ on the surgeon’s ear, the NAVIO robot reaches sound levels of 134.48 dB(C) compared to 128.98 dB(C) using the MAKO robot and 126.48 dB (C) using the CORI robot. All results are shown in Tables 1, 2.

**Table 1:** Results of LAeq of MAKO, NAVIO and CORI robot

Groups	Measured LAeq (dB(A))	Calculated LAeq (dB(A))	Recommended value of NIOSH for LAeq (dB(A))
MAKO	81.7	93.2	88
NAVIO	77.4	88.9	
CORI	77.9	89.4	

## Discussion

The most important finding of this study is that the LAeq was significantly higher during TKA using a MAKO robot compared to the sound levels of the NAVIO and CORI robot. A possible explanation could be the continuous noise generated by the ventilation of the robotic arm, even when the robot itself is not in use. The NAVIO and CORI robots, on the other hand, are small handpieces that do not require continuous ventilation, and therefore, do not generate any noise when not in use.

**Table 2:** Results of LCpeak of MAKO, NAVIO and CORI robot

Groups	Measured LC_peak_ (dB(C))	Calculated LC_peak_ (dB(C))	Recommended value of NIOSH for LCpeak (dB(C))
MAKO	117.5	134.4	140
NAVIO	123.0	129,0	
CORI	115.0	126.5	

The measured average sound level from each robot was below the above-mentioned recommended guidelines from NIOSH and the national guidelines from Germany and Great Britain. However, if calculating the average noise level exposure of the surgeon’s ear with formula 1.1, the LAeq exceeds the recommended values of the guidelines. It indicates that each robot, especially the MAKO robot, could be harmful to the surgical staff and lead to NIHL in the long term.

**Fig. 3:**
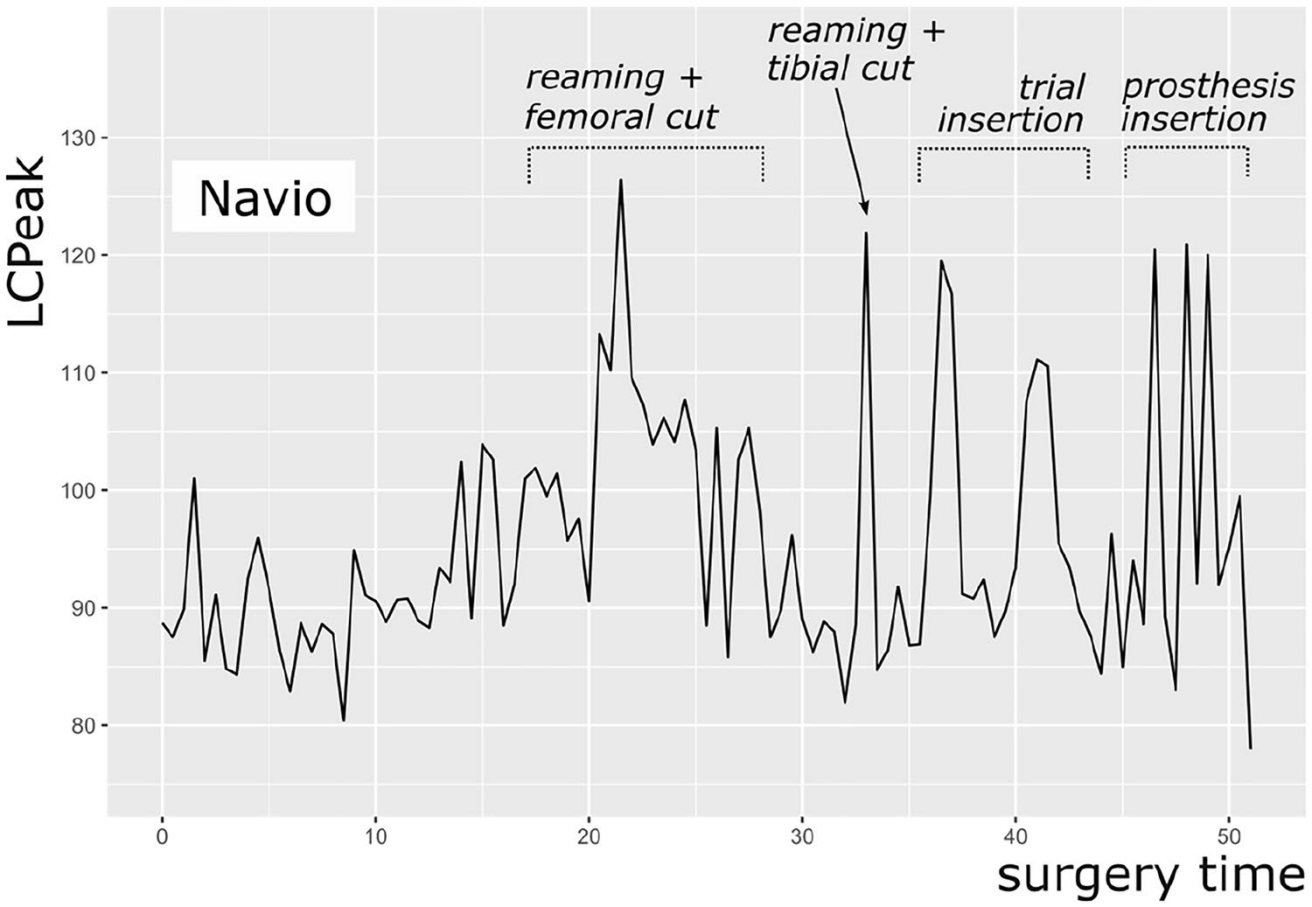
An exemplary course of LC_peak_ by NAVIO robot showing the volume of the main steps of surgery

**Fig. 4:**
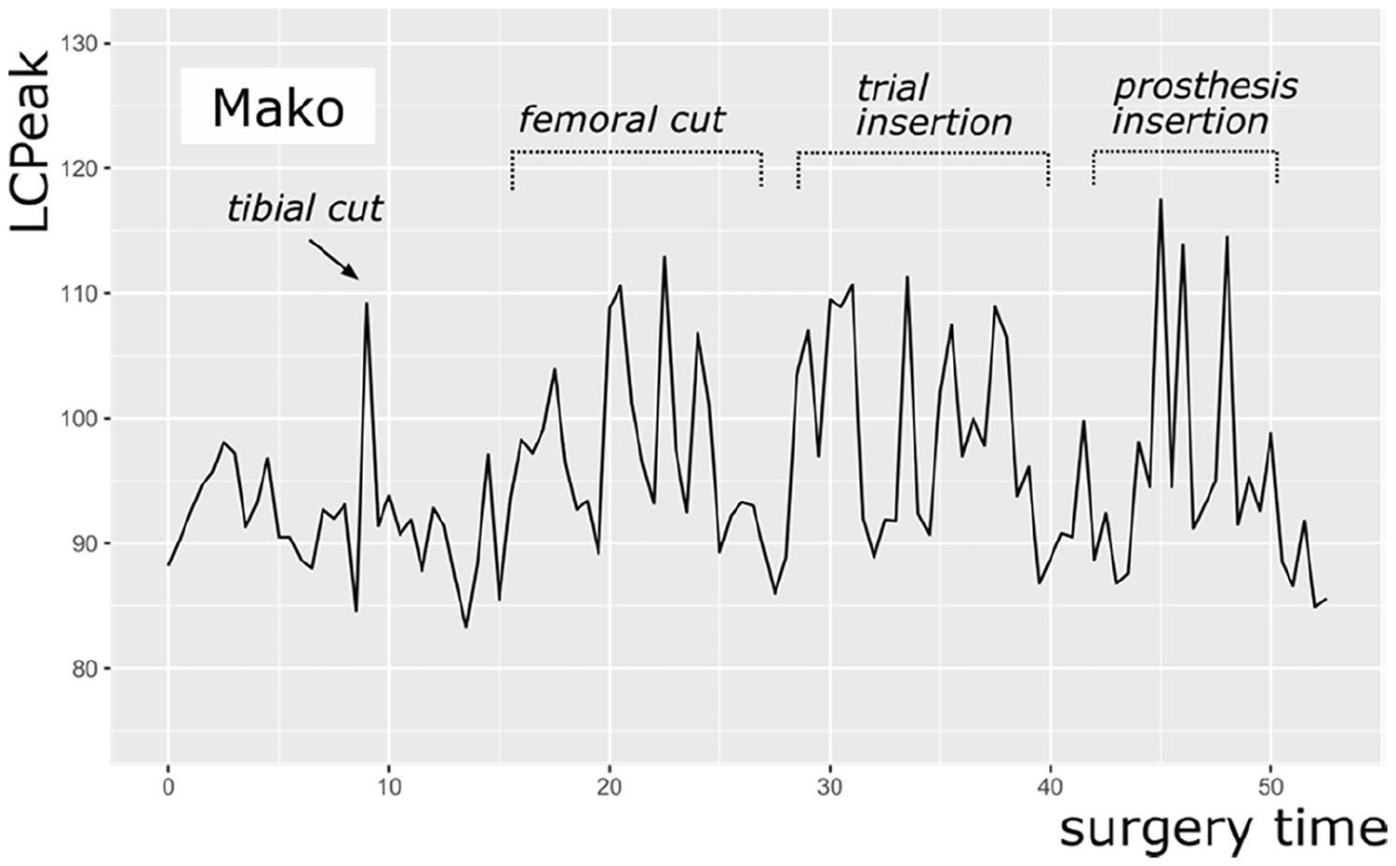
An exemplary course of LC_peak_ by MAKO robot showing the volume of the main steps of surgery

**Fig. 5:**
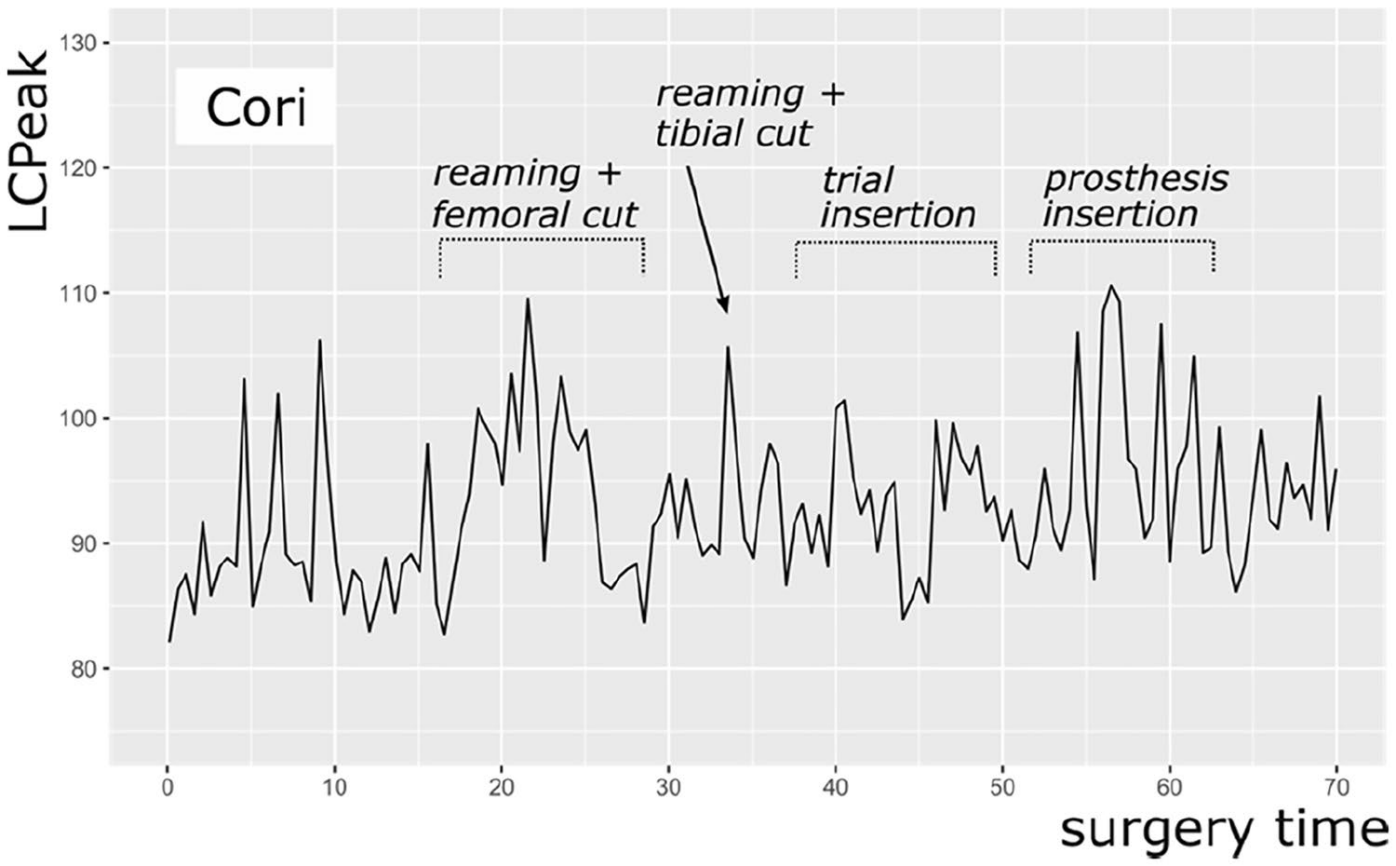
An exemplary course of LC_peak_ by CORI robot showing the volume the main steps of surgery

Furthermore, the intermittent use of high-power instruments, like the saw, mallet, or reamer, results in high impact noise levels measured by LC_peak_. The surgery with a NAVIO robot reached higher LC_peak_ (123.0 dB(C)) than the surgeries with a MAKO robot (117.5 dB(C)) and CORI robot (115.0 dB(C)). A possible explanation could be the reaming of the distal femoral cut with the NAVIO handpiece. Figures 3, 4 and 5 show exemplary courses of the LC_peak_ during surgeries with the three different robot systems. In the course of the NAVIO robot (Fig. 3), there are sound pressure peaks in the second third of the surgery, which could be due to the use of the NAVIO reamer. Compared to the course of the CORI robot (Fig. 5), no sound pressure peaks can be detected. It indicates that the reamer of the CORI handpiece does not generate these sound level peaks while reaming the femoral bone. This difference was significant (*p* = 0.005).

Another possible explanation for the peak sound pressures could be hammering the saw block device into the femur after reaming the distal femoral cut. The mallet strokes on metal generate significant noise. This step does not have to be performed in surgeries with MAKO robot. However, in surgeries with CORI robot, this step is also conducted using the same instruments as in surgeries with NAVIO robot, so that is unlikely the reason for the sound pressure peaks during the surgery with NAVIO robot.

When calculating the sound level exposure of the surgeon’s ear, none of the robots exceeds the recommended values for impact noise by the safety guidelines for noise level exposure set by NIOSH or the government of Germany and Great Britain. Nevertheless, high noise peaks are reached during surgeries with all robot systems, which are probably harmful to human hearing.

Other studies measuring sound levels of conventional TKAs obtained similar results regarding the noise levels. Palmer et al. measured an average noise level exposure during TKA of 77.2 dB(A), while the LCpeak was 134.4 dB(C) [14]. The survey by Love et al. demonstrated slightly higher values of noise exposure. Thus, an average noise exposure of 79.7 dB(A) and an LCpeak of 145.5 dB(C) were measured [6].

In conclusion, robotic devices do not result in additional noise exposure compared with conventional TKA. Nevertheless, both conventional and robot-assisted TKAs exceed the limits of the prescribed maximum values for LAeq of NIOSH and the national guidelines of Germany and Great Britain. Hence, the risk of NIHL in advanced age is at hand.

Regardless of the potential damage to human hearing, reducing noise in the operating theater could improve the surgical outcome. Engelmann et al. show in their study that an overall reduction of noise reduces postoperative complications and decreases the surgeon’s stress level [15]. To protect both the operating theater staff and the patient’s surgical outcome, the volume in operating rooms should be reduced.

Generally, there are several approaches to encounter high sound levels during surgeries. One possible way to reduce sound level exposure of the operating theater is the use of hearing protection. However, the impaired communication between the operating theater staff seems problematic as it might significantly decrease the surgery’s performance. Earmuffs with electronic noise cancelling would be necessary to prevent this, attenuating high noise levels but not impairing communication. However, wearing hearing protection would pose an additional risk to the surgical area’s sterility.

The use of modern instruments would be another option to reduce noise. Peters et al. demonstrated that the use of tip-oscillating saw blades resulted in a significantly lower noise exposure than conventional oscillating saw blades [16]. The LAeq of conventional saw blades, which were also used in the present study, was 93.1 dB(A), while the values of tip-oscillating saw blades were 84.4 dB(A) and 81.3 dB(A), respectively. Sydney et al. obtained similar results. They examined different saw blades on a porcine femur or tibia [17]. In their study, the tip-oscillating saw blades produced significantly less noise with 81.6 dB(A) than the conventional saw blades with 88.9 dB(A). Accordingly, the use of modern saw blades could help to alleviate noise exposure for operating theater staff.

Another potential problem due to noise pollution is the fact that it is common for surgical staff to perform both TKA and total hip arthroplasties (THA) on the same day. This study did not measure the noise exposure from THA. However, research concluded that THA produces even more noise and leads to a higher exposure of noise during a working day due to the reaming of the acetabulum and hammering the stem into the femur [6, 10, 14].

In contrast to the findings of the studies mentioned above, other studies could not find hazardous noise levels during TKA [18, 19]. Slaven et al. recorded TKA and THA sound levels and compared them with sound levels of arthroscopic surgeries. They did not find any sound levels above the NIOSH recommendations neither during THA nor during TKA. One potential reason for that could be the measurement technique using a mobile phone-based sound level meter placed in the breast pocket of the surgeon. In addition to a smartphone not being a calibrated measuring instrument, the sterile surgical gown above the mobile phone could have attenuated the measured sound levels.

### Limitations

In this study, noise level measurement could not be performed directly at the surgeon’s ear, compromising the surgical site’s sterility. Thus, the noise exposure of the surgeon had to be calculated. However, the comparison of our results with results from other studies shows that the calculating noise exposure is similar to directly measured values. Nevertheless, further studies would need to examine the differences between noise exposure at the surgeon’s ear and the measurement device.

Another point of concern is the small sample size. As it is assumed that noise exposure during surgery does not show severe variability, such a small sample number of the respective groups (MAKO, NAVIO, CORI) was used in the present study.
