# Correction to: Noise exposure during robot-assisted total knee arthroplasty

**DOI:** 10.1007/s00402-022-04477-3

**Published:** 2022-05-20

**Authors:** Tim Hönecke, Michael Schwarze, Matthias Wangenheim, Peter Savov, Henning Windhagen, Max Ettinger

**Affiliations:** 1 Department of Orthopedic Surgery, Hannover Medical School, Anna-von-Borries-Str. 1-7, 30625 Hannover, Germany; 2 Laboratory for Biomechanics and Biomaterials, Hannover Medical School, 30625 Hannover, Germany; 3 Institute of Dynamics and Vibrations, Leibniz University Hannover, Welfengarten 1, 30167 Hannover, Germany; 4 Hahnenstraße 13, 30167 Hannover, Germany

## Correction to: Archives of Orthopaedic and Trauma Surgery 10.1007/s00402-022-04454-w

The original version of this article unfortunately contained a mistake. The given name and family names of authors were interchanged except the name of Peter Savov.

The correct Names are (Family Name, Given Name):

Hönecke, Tim.

Schwarze, Michael.

Wangenheim, Matthias.

Windhagen, Henning.

Ettinger, Max.

The original article has been corrected.

